# "I am nothing": experiences of loss among women suffering from severe birth injuries in Tanzania

**DOI:** 10.1186/1472-6874-11-49

**Published:** 2011-11-15

**Authors:** Lilian T Mselle, Karen Marie Moland, Bjørg Evjen-Olsen, Abu Mvungi, Thecla W Kohi

**Affiliations:** 1 School of Nursing, Muhimbili University of Health and Allied Sciences, Dar es Salaam, Tanzania; 2 School of Nursing, Bergen University College, Bergen, Norway; 3 Centre for International Health, Bergen, Norway; 4 Department of Obstetrics and Gynaecology, Sørlandet Hospital, Kristiansand, Norway; 5 Department of Sociology and Anthropology, University of Dar es Salaam, Dar es Salaam, Tanzania

## Abstract

**Background:**

Despite the increased attention on maternal mortality during recent decades, which has resulted in maternal health being defined as a Millennium Development Goal (MDG), the disability and suffering from obstetric fistula remains a neglected issue in global health. Continuous leaking of urine and the physical, emotional and social suffering associated with it, has a profound impact on women's quality of life. This study seeks to explore the physical, cultural and psychological dimensions of living with obstetric fistula, and demonstrate how these experiences shape the identities of women affected by the condition.

**Methods:**

A cross-sectional study with qualitative and quantitative components was used to explore the experiences of Tanzanian women living with obstetric fistula and those of their husbands. The study was conducted at the Comprehensive Community Based Rehabilitation Tanzania hospital in Dar es Salaam, Bugando Medical Centre in Mwanza, and Mpwapwa district, in Dodoma region. Conveniently selected samples of 16 women were interviewed, and 151 additional women responded to a questionnaire. In addition, 12 women affected by obstetric fistula and six husbands of these affected women participated in a focus group discussions. Data were analysed using content data analysis framework and statistical package for the social sciences (SPSS) version 15 for Microsoft windows.

**Results:**

The study revealed a deep sense of loss. Loss of body control, loss of the social roles as women and wives, loss of integration in social life, and loss of dignity and self-worth were located at the core of these experiences.

**Conclusion:**

The women living with obstetric fistula experience a deep sense of loss that had negative impact on their identity and quality of life. Acknowledging affected women's real-life experiences is important in order to understand the occurrence and management of obstetric fistula, as well as prospects after treatment. This knowledge will help to improve women's sense of self-worth and maintain their identity as women, wives, friends and community members. Educational programmes to empower women socially and economically and counselling of families of women living with obstetric fistula may help these women receive medical and social support that is necessary.

## Background

Severe birth injuries cause life-long disabilities and poor quality of life. In low-income countries, between 15 and 20 million girls and women develop disabilities following obstructed labour every year [1]. These injuries could have been prevented if adequate, quality emergency obstetric care services had been accessible. Because of these gross inequalities, Millennium Development Goal 5 targeting maternal health has drawn attention to maternal mortality and morbidity. Recent studies have documented persistent lack of progress on this indicator in sub-Saharan Africa [2]. Obstetric fistula is a grave birth injury that, in the absence of surgical interventions, causes chronic physical and social disability, typically affecting poor women living in rural areas. This study focuses on women's experiences of living with obstetric fistula in the economic, social and cultural context of rural Tanzania. It raises issues related to the social interpretation of illness and suffering, and discusses the disability and social ostracism experienced by fistula sufferers as a major equity issue in public health.

### Obstetric Fistula: Magnitude and Scope

Obstetrical fistula is a defect in the genital tract connecting the vaginal or uterine cavity to the bladder, urethra, ureters, rectum or colon. It is acquired during the process of labour, usually when there is a delay to intervene a prolonged obstructed labour [3]. As a result, the compressed surrounding soft tissues are subject to necrosis, thus creating an open communication between the bladder and vagina (vesico-vaginal fistula-VVF) or vagina and rectum (recto-vaginal fistula-RVF), through which urine and or faeces leak [4-6]. Other types of fistula may occur due to cervical cancer, radiation therapy or injuries following surgery [7]. Studies on women affected by fistula in resource-poor countries have shown that socio-cultural and health system factors are associated with obstetric fistula. These factors include cultural beliefs and practices, limited decision-making power, illiteracy, low status of women, sexual inequality, malnutrition, and the lack of emergency obstetric care [8-14].

Estimates indicate that 2-3 million women live with obstetric fistula worldwide, the majority of whom are in Africa and Asia [15-17]. Moreover, it has been shown that in up to 83% of obstetric fistula cases, the baby is stillborn or dies within weeks after delivery [18]. In Africa, estimates indicate that between 30,000 and 130,000 new cases of obstetric fistula develop each year. In Tanzania, about 2,500-3,000 new cases of fistula occur annually [19]. Living with fistula has a profound effect on women's quality of life. Because of incontinence, families and communities tend to view women who live with fistulas as defective, and this largely influences how women experience living with it. These women have to cope with pain, discomfort, shame, depression, isolation, and stigma from the community, as well as from their own spouses and families [20,21].

To improve the quality of life of women living with fistula, surgical fistula repair is ideally performed. In 2001, 50 hospitals in Tanzania were performing fistula repairs and there were doctors with specialised training [22]. Fistula repair has shown very promising results, with rates of successful repair being between 85% and 90% [23,24] if performed by an experienced surgeon, with appropriate nursing care and prevention of complications [23,25]. Nevertheless, existing fistula repair resources are not sufficient in light of the increasing number of newly registered cases of fistula that occur in Tanzania every year [26]. In addition, many women still have some incontinence despite successful surgical closure of fistula, largely due to extensive urethral damage and scarring [27,28]. They do not always regain the nerve and muscle control needed to stay dry. Therefore, many women live with fistulas for years and have to face the agony associated with it.

### Focus of the study

In Tanzania, a few studies on obstetric fistula have explored social vulnerability through women's own accounts [29,30]. This study explored the physical, cultural and psychological dimensions of living with obstetric fistula. Moreover, we wished to study how these experiences and ideas affected their roles as women and wives. To increase our understanding of the social dynamics at work on the family and community level, this study also included the perspectives and experiences of men as husbands, as extended family members and as providers in poor households.

### Identity, disability and discrimination

This study draws upon concepts of identity, disability and discrimination. Jenkins [31], defines identity as ways in which individuals and collectivities are distinguished in their social relations with other individuals and collectiveness. Identity can be social or personal. Social identity is a set of persons marked by a label and distinguished by rules deciding membership and characteristic features or attributes. Social identity is mainly based on a person's knowledge that he or she belongs to a particular social category or group [32]. Personal identity is the self, as reflexively understood by the person in terms of his or her biography [33].

Disability on the other hand, is a social implication to the individual's physical condition or impairment. A person is viewed as disabled when he or she lacks ability to perform an activity in the manner that is considered normal for a human being because of a physical or mental impairment [34]. Disability is closely associated with identity, the way that you see yourself and how others look at you. Goffman's [35] analysis of 'spoiled identity' described how impairment can destroy ones identity because it is viewed by society as disabling.

In Erving Goffman's book, *Stigma: Notes on the management of spoilt identity*, he defined stigma as "an attribute that is deeply discrediting." His conceptualisation of stigma focuses on the public's attitude toward a person who possesses an attribute that falls short of societal expectations. Stigma and discrimination may be closely linked. The person with the attribute is "reduced in our minds from a whole and usual person to a tainted, discounted one." From a public health perspective, stigma or discrimination is associated with worsened health outcomes [36] and is thought to be linked to reduced self-esteem [37] and quality of life [38].

## Methods

### Study design and setting

A cross-sectional study with qualitative and quantitative components was conducted between October 2008 and February 2010. The study was both hospital- and community-based. The rationale for the study being done in the community was to obtain the opinion of women's husbands, who could not easily be accessed in the health facilities. The study sites included the Comprehensive Community Based Rehabilitation Tanzania (CCBRT) and Bugando Medical Centre (BMC) hospitals and Mpwapwa district. Mpwapwa was chosen because half (14) of the women who were interviewed at CCBRT hospital were from this district.

The CCBRT is a private non-governmental organisation (NGO) hospital and a major service delivery point for obstetric fistula repair located in Kinondoni district, Dar es Salaam. It has a fistula ward with 21 beds and a hostel, where fistula patients live while waiting for fistula repair. The BMC is a consultant and teaching hospital in Mwanza, the second largest city in Tanzania, located on the shores of Lake Victoria. It has 900 beds and a fistula ward with 70 beds. On average, 300 women with obstetric fistula are treated there annually. Lastly, Mpwapwa is one of the seven districts in Dodoma region in the central part of Tanzania. It constitutes three divisions; Mpwapwa, Kibakwe, and Rudi, with 84 villages. It is one of the poorest areas of Tanzania, with little in the way of light industry or cash crop cultivation. Most of the population are subsistence farmers, and approximately 90% of the people live in small rural villages. In 2002, the district had an estimated population of 254,500 and Dodoma region had a population of 1,735,000 [39].

### Data collection

The study used a number of methods of data collection, including semi-structured interviews, focus group discussions, and a questionnaire.

#### The qualitative study

##### Semi-structured interviews

The senior nurse midwife identified 16 women who were living with obstetric fistula who happened to be in the CCBRT fistula ward during the data collection period. The inclusion criteria were: admission into the hospital for obstetric fistula repairs before or after they had surgery; ability to speak Kiswahili; and agreeing to participate in the study. The purpose of the study and principles of confidentiality were explained to the informants. Thereafter, a convenient time for an interview was arranged. The first author conducted all individual face-to-face interviews in Kiswahili, which is the national language. An open-ended interview guide was used, with topics and probing questions focusing on background and experiences of living with obstetric fistula for each of the women surveyed. The guide was revised during the course of data collection to allow new emerging issues to be included. In each interview, the informant was the major speaker and the researcher was mainly a guide and a facilitator. The level of openness of the interviewees varied, but seemed to be generally good. All interviewees agreed to the use of an audio-recorder and interviews lasted between 45 minutes to 2 hours.

##### Focus group discussion (FGD)

Two FGDs with women living with obstetric fistula (n = 12) were held at CCBRT hospital. One FGD with husbands of women affected by fistula was held at Tambi village in Mpwapwa district. Husbands were identified through the Anti-Female Genital Mutilation Network project (AFNET), Mpwapwa branch. AFNET is an NGO that deals with campaigns against female genital cutting, life skills, and issues related to reproductive health. AFNET works closely with CCBRT hospital in identifying women living with obstetric fistula in the district, and arranges for their transportation to CCBRT hospital for fistula repair. The first author contacted AFNET because they knew some of the husbands of women affected by obstetric fistula. Husbands who agreed to take part in the study were recruited. The group included six informants of different ages and ethnicity. Discussions were moderated by the first author using the FGD guide that was centred on the husbands' experience of living with women affected by obstetric fistula. Discussions were held in Kiswahili, a language spoken by all informants. The moderator was assisted by two research assistants during the discussions, one taking notes and another making observations. Although the number of informants in all FGDs was small, they elicited valuable information. Each FGD lasted for 1-2 hours and was audio-recorded with permission from the informants.

#### The quantitative study

Prior to the main study, the close-ended questionnaire was piloted at Muhimbili National Hospital. For the main study following the pilot, a convenient sample of 151 women was selected from both the CCBRT and BMC. To ensure adequate numbers of women affected by obstetric fistula were obtained during the study period between July 2009 and February 2010; all women affected by obstetric fistula admitted in the fistula wards were asked to participate. Only those who provided informed consent were recruited and there was no woman who refused to participate. Two research assistants (a senior nurse midwife and a nurse teacher, both with experience in health research) collected data in Kiswahili, one in each hospital. However, in order to test the setting, the first author administered five questionnaires in each selected facility.

### Data analysis

#### The qualitative data

The qualitative data from both interviews and FGDs were transcribed verbatim and translated from Kiswahili to English. A different person back-translated two transcripts [40] to Kiswahili to ascertain the quality of translation. There were no significant differences between them. Transcripts were read several times and meaning units were extracted and condensed by shortening the original text, while maintaining the core meaning. These condensed versions were condensed further to codes and grouped into categories [41]. 'Member checks' [42] was used to increase credibility.

#### The quantitative data

Quantitative data entry and descriptive analysis were done using Statistical Package for the Social Sciences (SPSS) version 15 for Microsoft Windows. Descriptive analysis was done where frequencies and proportions were used to present findings. In addition, cross-tabulation and chi-square statistics were used to assess significant associations between variables. Findings from the husband FGDs and the quantitative study were used to enhance and broaden our understanding on how women experience living with obstetric fistula.

### Ethical considerations

Ethical approval to undertake the study was obtained from the Muhimbili University of Health and Allied Health Sciences (MUHAS) Research and Ethical Review Board (Ref. no. MU/RP/AEC/Vol.XII/57 and MU/DRP/AEC/Vol.XII/62). Further, the CCBRT and BMC hospitals granted permission for data collection. Informants gave their informed consent to take part in the study after receiving detailed information regarding the voluntary nature of participation and about confidentiality. All names used in this article are fictive.

## Results

### Socio- demographic characteristics

The 28 women interviewed in the qualitative study were between 17-50 years of age. Twenty-two of them were from rural remote areas of Tanzania. Among these, 14 were from central regions of Tanzania. Eight were illiterate and only four had education beyond primary. All informants were peasants, petty businesspersons or homemakers. Eleven informants were either divorced or separated, 10 were married, six were single and one was a widow.

Out of 151 women living with obstetric fistula in the quantitative study, the majority (39%) were between 21-30 years of age, and 18% were under 18 years of age. The overwhelming majority of women had low education: 97% were either illiterate or had only completed primary education, 18% were divorced, and 60% were still living with their husband/partner at the time of study.

### Experiences of living with obstetric fistula

Ten of the 28 women in the qualitative study lived with obstetric fistula for more than six years, whereas of 151 women in the quantitative study, about 25% lived with fistula for three years or more, and all (n = 4) women who had secondary education or higher lived with it for a year or less. There was a significant (p = 0.011) association between living alone and duration that women lived with fistula. The duration of the time that women had lived with obstetric fistula differed considerably (Table 1).

**Table 1:** The duration women lived with obstetric fistula

Duration of leaking	Number	Frequency (%)
< 1 year	86	57.0
1-2 years	28	18.5
3-6 Years	17	11.3
> 6 years	20	13.2
**Total**	**151**	**100.0**

**Figure 1:**
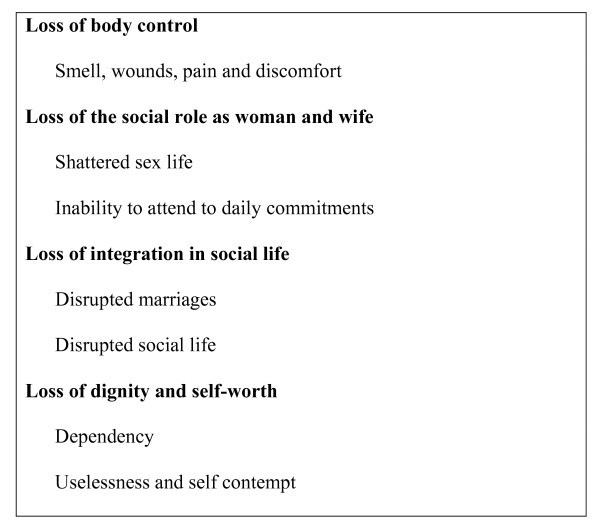
**Women's experiences of loss**.

In the analysis of the lived experiences from the perspectives of women affected by obstetric fistula, four themes emerged (see Figure 1) related to experiences of loss on the physical, emotional and social level: Loss of body control, loss of social role as a woman, loss of integration in social life, and loss of dignity and self-worth.

### Loss of body control

#### Smell, wounds, pain and discomfort

Continuous leaking of urine and or faeces was an extremely trying experience:

*"When you sleep and wake up all clothes are wet, when you work it flows on its own" *(Jane, 25 years old, lived with fistula for one year).

The smell, rashes, itching, peeling of the skin and sores were common experiences and part of everyday life. To some the pains were so severe that it hindered their daily activities and movements:

"*...the skin is so sore; sometimes I cannot even walk" *(Jane, 25 year old, lived with fistula for one year).

For all the women, the smell of urine was intolerable and a constant source of embarrassment causing women to withdraw from social life:

*"I always stay in my room due to strong smell from urine" *(Asha, 28 year old, lived with fistula for 2 months).

Another woman explained how she tried to contain the smell and in so doing causing more harm:

*"...I tried using water mixed with dettol to clean the vagina (douching), but eventually I ended up developing bruises and sores. I could not even walk" *(Saumu, 20 year old, lived with fistula for 6 months).

Owing to excessive leakage of urine and poverty, some women could not use cotton wool or sanitary pads. Instead, they resorted to using used plastic shopping bags 'rambo' to prevent urine from dribbling all the way down their legs. However, the plastic bags were very tough and rough on the skin as a result many developed bad sores:

"*...cotton pads are useless, they fall apart like water, 'kitenge' (a piece of cloth) is better, however even the kitenge itself does not last long. In less than a minute, it is soaked, because the way urine leak is as if a tap has been turned on. It reaches a time you wrap yourself with a plastic sheet 'rambo', yes like a baby you tie like this (demonstrates) but consequently you get bad wounds" *(Woman, FGD- CCBRT).

### Loss of the social role as a woman and wife

#### Shattered sex life

Living with fistula for years, and hence continuous leaking of urine and symptoms associated with it, obviously affected women's sex life. Only in rare cases could a woman with fistula continue having sex with her partner. Sexual abstinence was common and was experienced as a major loss:

*"...since I got this problem, we have not slept together up to now... and this is the most painful thing" *(Jane, 25 year old, lived with fistula for one year).

The pain of being seen as unclean and sexually undesirable was a shared experience:

*"...a lot of urine is coming out and my husband will not agree to have sex with me because it is dirty" *(Cathy, 35 year old, lived with fistula for 2 years).

The husbands presented the problem more as one of lack of sexual interest on the part of their wives:

*"...they (wives) don't like sex because a woman with fistula lives with wounds, therefore she will be in pain most of the time and she will not enjoy it" *(Husband, FGD- Mpwapwa).

However, they also reported lack of desire to have sex with their wives:

*"Firstly is the discomfort of leaking urine, the smell, and the soaked clothes which burns... yes...burns her genitalia, resulting in her developing wounds. ...again, as a man, trying to have sex with her; on the first day I did not feel well, it was distasteful and unpleasant all the way through" *(Husband, FGD-Mpwapwa).

Some women reported that their husbands sometimes had sex with them out of pity:

"*....perhaps twice in a month or at times once per week...when he looks at you, he feels sorry, but you never know now whether he has affairs" *(Woman, FGD-CCBRT).

Some husbands were worried about their wives becoming pregnant again and therefore decided to abstain totally:

*"...if we had sex could not she become pregnant again? For this reason I have never attempted, we are just living until today (laughs) I live with her nicely, she cooks, we eat" *(Husband, FGD- Mpwapwa).

Many of the women who still did not have any children wanted to have sex in order to get pregnant:

*"...I feel bad. I stopped having sex a long time ago. Perhaps if I was sexually active I could have had another child, now that I don't do it, how can I get a baby?" *(Lisa, 43 year old, lived with fistula for 20 years).

Women who already had children had different concerns:

*"I am exhausted and tired of washing clothes. If you sleep with your husband, the following morning you have to wash your clothes and those of your husband, and you will cause people to start running away from your husband because of the smell of urine" *(Cathy, 35 year old, lived with fistula for 2 years).

Husbands' experience of living in the same room with a woman with an untreated fistula was very unpleasant and trying, and as a result, many could not cope with the situation:

*"...frankly for those of us who have lived with those women (quiet moment), it is very tough. You cannot sleep until morning; you will be forced to wake up at night to change beddings" *(Husband, FGD- Mpwapwa).

#### Inability to attend to daily commitments

In addition to the leakage and the pain and discomforts of the wounds, women tended to experience general body weakness, which reduced their capacity to carry out their day-to-day responsibilities. Because of this experience, some women did not have the courage to go back to their homes after they got the fistula:

"*...I could not return to my husband because I am unable to carry out my daily duties. I have just been staying at my mother's house. ...I have not been able to work, so if I went to live with my husband that could have caused problems I would not have been able to wash his clothes nor mine. Until now, I only clean my toilet rags. My sister's daughter washes my clothes.*

*Misunderstandings could have arisen, that is why I did not return" *(Asha, 28 year old, lived with fistula for 2 months).

Many women spoke of their inability to carry out domestic chores or earn a living through farming, business, or employment. Some women were not allowed to cook for the family as they were judged as dirty or unclean:

*"...I am not cooking, yes, because when I cook, others, including my husband do not eat. They see it as dirt. ... if I prepared food, he (husband) would not eat, thereafter he started to cook food himself" *(Lisa, 43 year old, lived with fistula for 20 years).

However, it was clear from the discussion with the husbands that a woman with this problem, in special circumstances, could be allowed by their husbands to cook, particularly if they have children:

"...*because of her condition, she cannot farm, but she can cook, and since she has children she has to cook" *(Husband, FGD- Mpwapwa).

Consequently, because she could not farm, the work force of the household was reduced:

*"...we used to farm together, but now I have to do it alone. She cannot stand for a long time and there must be water around so that she can clean and wash her clothes" *(Husband, FGD- Mpwapwa).

The loss of ability to work was seen as a great obstacle to progress:

*"...Before, I was able to earn a living on my own. I was working in other people's houses as a maid. For now I cannot work for anybody because I am afraid of staying in peoples houses...I am just afraid... I am afraid of soaking other people's beds" *(Miriam, 28 year old, lived with fistula for 10 years).

The feeling of being dirty due to leaking urine and the smell contributed to the women's failure to continue working:

*"... I had a petty business stall. I was selling doughnuts, but now I cannot live that kind of life...I cannot sit for long...again, if you prepare doughnuts while you are leaking urine who will buy and eat them?" *(Gloria, 32 year old, lived with fistula for 19 years).

### Loss of integration in social life

#### Disrupted marriage

Women living with obstetric fistula frequently described rejection by their husbands and family members. Women explained that the problem of fistula had separated them from their husbands because it limited their ability to fulfil marital roles. Eleven of the 28 women in the qualitative interviews and 18% of the women in the quantitative study were not living with their husbands during the data collection period. They were either divorced or separated. Women felt that their inability to get pregnant and bear children played an important role in them being abandoned:

*"...because I am leaking urine, I am useless, I have no value. If I did not have this problem, my husband would not have abandoned me. But my husband left me because I am leaking urine and I would not bear a child for him" *(Doto, 28 year old, lived with fistula for 12 years).

Some women were forced to go back to their parents, and for those who were not divorced, some lived in different houses or rooms. One woman remembered her husband telling her: *"I cannot tolerate and wait for you to be healed"... I feel that the inside of me is rotten" *(Gloria, 32 year old, lived with fistula for 19 years).

Other women stayed in the hospital seeking treatment for a long time. When they returned home, they found their husbands already remarried. Some husbands did not hesitate to say openly in the FGDs that they left their wives because of the leaking:

*"....yes I left her for good...we had to separate, I have two houses, she has her bed and her house and I have my bed and my house (laughs). We are like a brother and sister now" *(Husband, FGD- Mpwapwa).

There was the consensus among the husbands in the FGDs that a man who continued living with his wife after developing fistula and leaking had to have another woman, and that the wife should understand and accept this. As one man pointed out:

*"... I had to have another woman. Given the situation she was in, of leaking urine, she agreed; saying it is ok, just look for one to be with...yes she had to accept. If she refused, I could have left her" *(Husband, FGD- Mpwapwa).

Stigma surrounding the problem of fistula contributed to the husbands' decision to abandon their wives. Some husbands reported being ridiculed in the community:

*"...the community started laughing at me saying that it is better I divorced my wife because she was leaking urine and that she cannot deliver again" *(Husband, FGD- Mpwapwa).

Also in-laws could insist on divorce:

*"When I returned from the hospital, my sisters-in-law started saying, "Our sister-in-law is damaged and smells". They used to tell my husband; ..."how could you live with a woman who is leaking urine?" Later on, they called a family meeting and a decision was made that my husband should divorce me. They called my grandma, and told her ... "grandma, we do not want your granddaughter because she has this problem of leaking urine. We want our son to marry another woman..." *(Doto, 28 year old, lived with fistula for 12 years).

#### Disrupted social life

Women also experienced loss of contact with their friends, parents, and relatives:

*"...I used to have real friends in the past, but the current friendship is just the kind of asking "how are you today, I am ok" that is it. I have no close friends" *(Amina, 35 year old, lived with fistula for 18 years).

Although women described the experience of being isolated from friends and relatives, husbands in the discussion reported that as long as you live with a woman who is leaking urine, the 'whole family' is isolated and is regarded as being unclean:

*"...the community will isolate the whole family. They will not ask for water, salt, flour, or anything from your house ...they cannot eat dirt. The house is just yours. Even utensils they will not ask from you nor will they let you borrow theirs... (mmh*)" (Husband, FGD- Mpwapwa).

Women reported stopping going to social gatherings such as funerals, church, mosque, parties, and visiting friends and families.

*"... also the urine which leaks freely, people look at you and you feel ashamed, even if there is a funeral in your family, you go to the graveyard, after that you go back to sleep at home because how could you sleep in other peoples houses with the problem of urine? You wake up smelling. You just return to sleep at home...as long as they saw you it is ok" *(Women, FGD-CCBRT).

The experiences of women keeping a distance from others were not only linked to being devalued and excluded by other people. According to husbands, women continuously dripping of urine also tended to distance themselves from their own family:

*"... my wife could never stay with me for more than 15 minutes or so" *(Husband, FGD- Mpwapwa).

*"...at times even sitting with her children is difficult. She has turned into one who hides and runs away from others, sits on one end of the house, alone" *(Husband FGD- Mpwapwa).

### Loss of dignity and self-worth

#### Dependency

Since the women living with obstetric fistula could not get involved in economic activities of any kind, they became more dependent on others. Women who used to earn money on their own felt particularly bad about being dependent on their husbands. The husbands talked about this state of dependency as marginal. They explained that it was like losing the status of an adult person and being relegated to the status and role more similar to that of a child:

*"...the problem is that fistula affects the family economy in general. My wife is not involved in any economic activity. She spends just too much time changing pads, she cannot sell doughnuts, she cannot sit to chat with her friends, she cannot be involved in group development activities, she has been reduced to a stage... living like a child carried on her mother's back" *(Husband, FGD-Mpwapwa).

The women also associated their dependency with a child's status, as one woman described her situation:

*"...I have turned back into being like a baby. I have to depend on my parents or relatives and neighbours" *(Gloria, 32 year old, lived with fistula for 19 years).

#### Uselessness and self-contempt

Many women hence were concerned that they had no role to play in family or community life. The feeling of being useless seemed to be pervasive and many struggled with self-contempt:

*"...I feel hopeless and useless; I even lost the baby" *(Gloria, 32 year old, lived with fistula for 19 years).

Also in the FDG with husbands, the uselessness of the woman with fistula came out strongly:

"She is not productive; there will be no children and no sex... (mmm)... we live like a brother and sister" (Husband, FGD- Mpwapwa).

A live baby would have eased the sense of uselessness:

*"It could have been better if I had my baby alive because if you have a problem but you still have your child" *(Gloria, 32 year old, lived with fistula for 19 years).

The women were also deeply concerned about their inability to keep clean and look neat. They wrapped themselves in old '*khangas*' or '*kitenges*' (types of materials with specific prints) in order not to ruin their best clothes:

*"...as a woman you need to be clean and dress nicely, things like underwear, but if you dress, it immediately soaks" *(Lisa, 43 year old, lived with fistula for 20 years).

For some, the feeling of being insignificant was so intense that they did not feel complete as women. Cathy, who had lived with fistula for two years said,"*...I am nothing, I feel like a child".*

Devaluation as a woman and as a human being was part of the experience of living with obstetric fistula, as one woman explained:

*"... don't be fooled that they love you. No, people will love you when you are clean but now how will you be loved... In fact if you have a problem like this nobody will value or love you" *(Woman, FGD- CCBRT).

## Discussion

### Reflection of the method

To better understand women's experiences of living with obstetric fistula, a mixed quantitative and qualitative method was used. In the study, women affected by obstetric fistula were recruited from CCBRT and BMC hospitals. In this case, it is likely that women who managed to seek care were those who received support from their relatives and communities, whereas those unsupported were less likely to come to the hospital. They may also have been suffering with severe negative experiences of living with fistula. It is also possible that selection bias was introduced by asking the AFNET project coordinator to identify husbands in the community because they may have selected those who were well known to them and who were more positive about the project. However, due to inaccessibility of this study group because husbands do not usually accompanying their wives to the hospital for treatment, the authors had little option other than to access them through this entry point. The direct quotes of women affected by obstetric fistula and those of their husbands are presented to allow the reader ascertain the validity or dependability of the study findings. The experiences of women living with obstetric fistula in this study are relevant to experiences of other women who live with it in other countries of sub-Saharan Africa, which have similar social-economic context especially in terms of the implication of these experiences.

### Multiple losses

The women living with obstetric fistula experience a deep sense of loss that affected them as women and wives. This study has described loss of body control, loss of the social role as woman and wife, loss of integration in social life, and loss of dignity and self-worth. The impairment caused by fistula produces disabilities that prohibit social participation and significantly reduce their possibilities to achieve their life goals. It bans women from having intimate relationships with their husbands, from giving birth to children, from establishing and maintaining social networking, from income generating activities, and from owning property. The following discussion focuses on how these losses on the part of the woman are signified as failures of motherhood, reproduction, sexuality, and marriage and how, in the social and cultural context of rural Tanzania, these 'failures' produce a 'spoilt identity' [35] and, in fact, deprives women with fistula of their identity as women.

#### Failure of motherhood

Women in Tanzania, as in many sub-Saharan African communities are expected to assume domestic chores, marry, and have children [43]. They acquire value and status from their husbands and society through their ability to bear children and fulfil their roles as women and wives [44]. It is the role as mother and wife that is the major source of respect and self-esteem [43]. Motherhood is located at the very core of a woman's identity. Her ability to bear and rear children defines her position in the kinship group and in the community. It is through giving birth to children and producing offspring to the clan that women slowly attain a position in the kinship group and in the community in general. A woman who has not given birth remains a girl and is excluded from the community of women and mothers. Studies [45,46] have documented that the experiences of women living with fistula who had children before they got obstetric fistula were relatively better than those who did not have children. Their husbands commonly let them stay, cook, and assist in raising their children even if the husband opted to have another woman. Some women consistently mentioned that it could have been better if they had children. Husbands' interest in keeping their wives seems to be closely connected to their children. Further, women commonly get access to resources through men as fathers, husbands, and sons. Without giving birth to male children, a woman may be left destitute in her old age. A barren woman is a woman who is commonly seen as useless, without any purpose. There will be nobody to replace her in life and for whom she will be an ancestor, and such a woman is devalued by the society and labelled with verities of stigma.

#### Failed sex life

The disabilities that women acquire from their inability to control the leaking of urine, the smell, the sores, and the infections make them unable to satisfy their husbands sexually or bear children. Since they are not capable of carrying out their ascribed social roles, they are less valued as wives, as well as members of a family and the greater community. In this study, women had strong negative feelings over their inability to have sex with their husbands and partners, largely because of their need to have children, as well as their need to reaffirm intimacy and bonding. Husbands on the other hand, explained that their wife's status has changed because of inability to have sex with them. During discussions, husbands emotionally said that they are now living with their wives as brothers and sisters. Healthy sexual life is the source of children and family bonding, and lack of it contributes to women's loss of self-esteem about confidence in their womanhood [47]. This is demonstrated by the higher rates of separation and divorce that appear common among women living with obstetric fistula [24].

#### Failed marriage

Illustrations of marital experiences of poor women living with obstetric fistula from this study suggest that these women feel they have been degraded to the status of a servant or divorced. Commonly if a woman has children, her husband would accept her staying on the compound, but would no longer treat her as a wife. Women were not allowed to cook, were forced to eat alone, live in separate rooms or beds, and often asked to return to the home of her parents. Women from this study who kept their marriages claimed that they did not have any intimate relationships with their husbands and thus considered themselves more like servants because they could not assume their normal roles as wives. Many (11/28) women interviewed were divorced or abandoned by their husbands due to obstetric fistula, which is consistent with previous studies by others [15,24,29,46,48-51]. The qualitative finding however, differed from the quantitative findings where 82% of women were still married during the study period and among them 70% lived with fistula for less than a year. A similar finding was noted in another study conducted in Tanzania, which indicated that the majority of women with obstetric fistula sustained their marriages [30]. Women are less likely to have their husbands' support when they suffer from fistula for a long period [50]. Moreover, maintaining marriage or living under the same roof does not necessarily imply that there are actual marital relationships between the couple [52].

It was further revealed that some husbands remain supportive of their wives in spite of the extra demands put on them and their unpleasant experiences of sharing a household with women leaking urine and or faeces. Few women reported receiving emotional, material, financial, and informational support from their husbands, but economic dependence made them accept unkind conditions in order to be retained and to be able to continue living in the family household. Women had to accept their husbands having a 'nyumba ndogo' (concubine) and extramarital relationships. This experience underscores the importance women place on keeping their marital status and of having children.

#### Failing social network

Women living with obstetric fistula experienced lack of invitation to participate in the social economic activities and thought that it was largely because of the bad smell, which prohibited other people to interact with them. Many lost their jobs and could not gather with the rest of the society in social events. This is in agreement with results found by others [24,29,30,48,53]. As a community member in Tanzania, culturally it is a norm and a commitment to take part in communal events such as wedding and funeral ceremonies. Failure to fulfil these social obligations is considered a serious breach on the basic social principle of reciprocal relationships and may lead one to be rebuffed by the community.

#### Family stigma

This study reveals that discrimination resulting from fistula affect not only the individual woman living with obstetric fistula, but also the whole family. Sharing and getting involved in other people's life events such as weddings and funerals are culturally important and essential in maintaining social networks in rural Tanzania. As shown in this work, neighbours avoid asking the family harbouring a woman with fistula to share anything including household items and vice versa, since the entire family is regarded as unclean and contaminated. The family is commonly not invited to take part in social events and is thus excluded from arenas where social relations are established, confirmed, and reinforced. Families become practically ostracised from the society. Some women reported that it was better for them to live in a separate house or room away from their husbands in order to spare their husbands from similar discrimination by the community. Furthermore, as has also been shown by others [49], it was not uncommon for families and friends to persuade husbands to break up with their affected wives and marry a new one who can give birth to children.

#### Spoilt identity

The individual, family, and social-cultural experiences women living with obstetric fistula go through fundamentally destroy these women's identity. The failures of the women to control urine and/or faeces, maintain their marriages, bear children, or participate in social economic activities make them lose their identity as women, wives, friends, and community members. They tend to see themselves as worthless, incomplete, and compare themselves with children. Adulthood is marked largely by not only managing one's emotions but also through being able to control body functions. Losing control of bodily functions is embarrassing as an adult. The cultural expectation of womanhood is embodied in the experience of the individual woman and produces shame and feelings of guilt. With an awful smell from the leakage of urine or faeces, women are unable to keep themselves clean and attractive. In Tanzania as in many parts of the world, a woman's beauty is associated with not only cleanliness, neatness, and sweet smell, but also with the capacity to assume domestic, marital, and social roles. Women living with obstetric fistula are deprived of all these attributes. This study, and others [8,13], show that many women living with obstetric fistula reside in rural areas with limited or no water supply, indicating that it is extremely difficult to keep clean. Moreover, because they cannot work and earn a living by themselves, they are unable to get cash to buy scented soap and lotion that could help control the smell. As one woman put it, "How can you be a woman with this condition of leaking urine and smell?" Nearly all women remained secluded indoors, away even from their families, friends, and the community. This tendency of women to isolate themselves was confirmed by husbands who confided that their wives were avoiding them because of feeling ashamed. These findings are in keeping with other studies [24,29,30].

#### Equity

The physical impairment and the social exclusion experienced by women living with obstetric fistula have a profound impact on their quality of life. The DALY (disability adjusted life years) [54] evaluation of the health burden associated with maternal ill health including obstetric fistula shows that the years of life lost due to disability is huge considering that the majority of women affected by obstetric fistula are still early in their reproductive phase of life [55-57]. Obstetric fistula does not hit randomly. The vast majority of women affected by obstetric fistula in this study constituted a socially weak group even before their birth injury. They were poor, uneducated, mostly young, married early, and lived in remote, rural, and poor resource areas with little or no access to emergency obstetric care. In addition, women who live with obstetric fistula are those who cannot easily travel to hospital facilities because of long distances, high costs of transportation, or lack of decision-making power in the family. Fistula reduces women's ability to work, and if in addition they do not have the support of their husbands and relatives, they could be driven deeper into poverty. This socially marginal background and status of women suffering from fistula has also been shown in other studies in Tanzania, as well as in other sub-Saharan African countries [24,58]. As documented in this work, the women's physical and social disability due to the injury pushes them further into marginalization, making them vulnerable to social exclusion and discrimination, as also noted elsewhere [6,22]. Obstetric fistula is a major equity issue both in the way it targets the poor and how it reduces quality of life.

## Conclusion

Living with fistula is associated with experiences of multiple losses that range from physical and emotional to social. These physical and social experiences of living with obstetric fistula have negative impact on women's identity and quality of life. Obstetric fistula represents a major equity problem that can only be addressed through improved access to social and economic development for girls and women in general, with ensuing better access to quality obstetric care. Despite the psychological and social sufferings of not having children, the women are unaware of the possibility that after fistula repair they may be able to be mothers again. Society at large needs to understand issues associated with occurrence and management of fistula and the potentially positive reproductive prospects after treatment. This may greatly help to improve the women's sense of worth and maintain their identity as women, wives, and friends in the society, and hence improve their quality of life. Educational programmes to empower women socially and economically and counselling of families of women affected with obstetric fistula may help women living with fistula in receiving necessary medical and social support.

## Competing interests

The authors declare that they have no competing interests.

## Authors' contributions

LTM was responsible for the study conception, the design, organisation and collection of qualitative data and supervised quantitative data collection. LTM, TWK and KMM performed data analysis and interpretation. LTM drafted the article. TWK, KMM, BEO and AM made critical revisions of the article. All authors were involved in the discussions during the planning and follow-up phases of the study at The Gender, Generation and Social Mobilization (GeSoMo) - NUFU workshops. All authors read and approved the final manuscript.

## Pre-publication history

The pre-publication history for this paper can be accessed here:

http://www.biomedcentral.com/1472-6874/11/49/prepub
